# Methodological research priorities for data sciences: Report from The International Methodology Consortium for Coded Health Information (IMECCHI)

**DOI:** 10.23889/ijpds.v1i1.389

**Published:** 2017-04-19

**Authors:** Rachel Jolley, Danielle Southern, Hude Quan, William Ghali, Bernard Burnand

**Affiliations:** 1 IMECCHI, The Methods Hub, University of Calgary; 2 CHUV- University Hospital of Lausanne

## Objectives

The vast amount of data produced by healthcare systems both structured and unstructured, termed `Big Data' have the potential to improve the quality of healthcare through supporting a wide range of medical and healthcare functions, including clinical decision support, disease surveillance, and population health management. As the field of big data in healthcare is rapidly expanding, methodology to understand and analyze thereby enhancing and optimizing the use of this data is needed. We present priorities determined for future work in this area.

## Approach

An international collaboration of health services researchers who aim to promote the methodological development and use of coded health information to promote quality of care and quality health policy decisions known as IMECCHI -proposes areas of development and future priorities for use of big data in healthcare. Thematic areas were determined through discussion of potential projects related to the use and evaluation of both structured /codeable and unstructured health information, during a recent meeting in October 2015.

## Results

Several themes were identified. The top priorities included: 1) electronic medical record data exploration and utilization; 2) developing common data models and multimodal /multi-source databases from disparate sources development; 3) data quality assessment including developing indicators, automated logic checks and international comparisons; 4) the translation of ICD-10 to ICD-11 through field-testing 5) Exploration of non-physician produced/coded data; and 6) Patient safety and quality measure development.

## Conclusion

A list of expert views on critical international priorities for future methodological research relating to big data in healthcare were determined. The consortium's members welcome contacts from investigators involved in research using health data, especially in cross-jurisdictional collaborative studies.

